# New Frontiers in Oncological Imaging

**DOI:** 10.3390/tomography9040105

**Published:** 2023-07-07

**Authors:** Chiara Zanon, Alberto Crimì, Emilio Quaia, Filippo Crimì

**Affiliations:** 1 Department of Medicine-DIMED, Institute of Radiology, University of Padova, Via Giustiniani 2, 35128 Padova, Italy; chiara.zanon.5@studenti.unipd.it (C.Z.); emilio.quaia@unipd.it (E.Q.); 2 Orthopedics and Orthopedic Oncology, Department of Surgery, Oncology and Gastroenterology DiSCOG, University of Padova, Via Giustiniani 2, 35128 Padova, Italy; alberto.crimi@aopd.veneto.it

The more that advances in the medical field are capable of targeted treatments, the more imaging should be tailored to patients. In this setting, the possibility of integrating artificial intelligence (AI), new techniques, and hybrid imaging modalities with standard imaging could represent a unique tool to extract as much data as possible from diagnostic images, thus differentiating the type of disease affecting patients and leading them toward the best therapeutic path [1,2].

In this Special Issue focused on the imaging assessments of cancer, from diagnosis to treatment [3], we had the opportunity to demonstrate much interesting research in the field of oncological imaging, embracing different subspecialities.

We will try to briefly summarize the main results of these papers.

Standardized reporting systems are taking up increasingly more space, reducing the variability in reports and improving the precocious detection of small lesions. In multi-parametric prostate MRI, the PI-RADS system has been endorsed by principal radiological societies and it was shown to improve the accuracy of MRI significantly for prostate cancer detection.

In their study, Sauck et al. assessed the capability of multiparametric MRI with the PI-RADS v2 scoring system and the capability of using the volume of lesions to detect prostate cancer, using MRI/trans-rectal-US-fusion biopsies as a reference standard. A total of 157 patients underwent MRI and targeted biopsies and the presence of prostate cancer was determined using the International Society of Urological Pathology (ISUP) grading system. The results indicated that 24% of the PI-RADS 3 lesions, 36.9% of the PI-RADS 4 lesions, and 59.5% of the PI-RADS 5 lesions were neoplastic. The volume of the lesions was significantly correlated with ISUP grades. The authors concluded that the PI-RADS v2 score and lesion volume were associated with the presence and clinical significance of prostate cancer, but that there was not an insignificant number of false positive findings [4].

Conventional cancer imaging approaches are based primarily on qualitative assessments, which could overlook some valuable information that can be contained in the images. Using textural analyses (TA), it is possible to elaborate on raw data provided by imaging by mean of US, CT, MRI, and PET, and to obtain quantitative parameters that are not normally appreciated by the human eye [5]. TA is a non-invasive imaging tool with great potential to predict pathological features, response to therapy, and prognosis for many tumors. Textural features showed similar or even stronger accuracies when detecting cancer compared with the qualitative evaluation of expert radiologists [6]. The correlation between radiomics data and clinical reports, pathology, histology, and genetic information could provide a global view of tumor biology. Several case studies have demonstrated the efficacy of radiomics texture analyses on CT scans for tumor diagnosis [7].

Crimì et al. investigated the relationship between contrast-enhanced CT TA and KRAS, NRAS, BRAF, and MSI mutations in patients with colon cancer. TA parameters were extracted from CT scans and compared between patients with wild-type and mutated genes. The results revealed statistically significant differences in four parameters between the microsatellite stable (MSS) and microsatellite instability (MSI) groups. These findings suggest that CT TA could potentially aid in identifying MSI in colon cancer by using pre-treatment contrast-enhanced CT scans [8].

Another field of research that has shown promising results in the oncological field is hybrid imaging, mainly driven by the introduction of new radiotracers and PET/MRI scanners.

Ince et al. determine whether 18F-FDG PET/MRI could improve clinical response assessments compared to MRI for nonoperative management in patients with rectal cancer undergoing total neoadjuvant therapy. PET/MRI showed a 100% accuracy in assessing complete responses compared to the 71% accuracy of MRI alone. The authors suggested that PET/MRI can improve residual disease detection after total neoadjuvant therapy and provide additional value for restaging and surveillance during watch-and-wait protocols in patients affected by rectal cancer [9].

Bodapati et al. presented two cases of intra-orbital metastases in patients affected by estrogen receptor-positive breast cancer studied with 18F-fluoro estradiol (18F-FES) PET/CT. Although rare, orbital metastases can significantly affect the visual acuity and quality of life, particularly in patients with breast cancer. The 18F-FES PET/CT technique is a novel estrogen receptor-specific radiotracer that provides a more precise assessment of the intracranial and infraorbital regions in estrogen receptor-positive cancers by minimizing the physiological background activity. The cases presented and a review of the literature demonstrated that 18F-FES PET/CT had a higher sensitivity than traditional 18F-FDG PET/CT in detecting orbital metastases in breast cancer [10].

Imaging has a pivotal role even in the diagnosis of secondary hypertension, especially by endocrinological causes.

Tizianel et al. emphasized the importance of individualized approaches for Primary Aldosteronism (PA) assessments. PA is a common cause of secondary hypertension and is associated with a high risk of cardiovascular and cerebral diseases. Classification of PA subtypes (unilateral or bilateral) is essential to determine the most effective treatment approach (surgical or medical). The authors presented five clinical cases representing different PA subtypes, highlighting personalized diagnostic and therapeutic processes tailored to each patient’s needs, medical history, and preferences [11].

Images can also guide biopsies, especially CT in lung lesions, allowing precise sampling and diagnosis.

Baratella et al. evaluated the diagnostic accuracy of CT-guided needle biopsies for primary lung malignancies in 350 thoracic biopsies. From this large studied sample, the authors concluded that CT-guided core needle biopsies are a minimally invasive, highly accurate, and safe procedure for diagnosing lung cancer, since it showed a 98.87% accuracy and only three patients experienced major complications after the procedure [12].

Finally, imaging could represent a valuable tool in identification of nodal metastases, especially in gastro-intestinal tumors. In addition, the accuracy of the identification of metastatic lymph nodes is suboptimal and thus different criteria and types of measurements could be used to enhance the diagnostic capacity of imaging.

Crimì et al. investigated the most accurate CT dimensional criteria for identifying metastatic lymph nodes (LNs) in patients with gastric cancer during preoperative staging. Various measurements, including the short axis (SA), the volume, the SA/long axis (LA) ratio of the largest LN, the sum of the SAs of all LNs, and the mean SA/LA ratio, were analyzed and compared with the presence or absence of LN metastases confirmed through histopathology. The sum of SAs demonstrated the highest area under the ROC curve (AUC) in the per-nodal group analysis, with a sensitivity and specificity of 62.4% and 72.6%, respectively, using a cutoff of >8 mm. In the per-patient analysis, the sum of the SAs of all LNs in the locoregional nodal groups yielded the best AUC, with a sensitivity and specificity of 65.6% and 83.7%, respectively. The authors concluded that the sum of the SAs of all LNs in staging CT scans is the most reliable predictor for both metastatic invasion of the nodal group and the presence of metastatic LNs in patients with gastric cancer [13].

In conclusion, oncological imaging is evolving rapidly, and these advancements are promising and could improve tumor diagnoses and treatment outcomes.
